# *Heatr9* is an infection responsive gene that affects cytokine production in alveolar epithelial cells

**DOI:** 10.1371/journal.pone.0236195

**Published:** 2020-07-17

**Authors:** Christopher J. Stairiker, Marjan van Meurs, Leticia G. Leon, A. A. Brouwers-Haspels, Laurine Rijsbergen, Yvonne M. Mueller, Peter D. Katsikis

**Affiliations:** 1 Department of Immunology, Erasmus MC, University Medical Center Rotterdam, Rotterdam, The Netherlands; 2 Department of Microbiology and Immunology, Drexel University College of Medicine, Philadelphia, Pennsylvania, United States of America; 3 Department of Virology, Erasmus MC, University Medical Center Rotterdam, Rotterdam, The Netherlands; Emory University School of Medicine, UNITED STATES

## Abstract

During infection, viruses enter susceptible host cells in order to replicate their components for production of new virions. In the process of infection, the gene expression of infected cells undergoes changes because of the production of viral components and due to the host response from detection of viral products. In the advent of RNA sequencing, the discovery of new genes and their functions in the host response generates new avenues for interventions in the host-pathogen interaction. We have identified a novel gene, *Heatr9*, as a virus and cytokine inducible viral responsive gene. We confirm *Heatr9*’s expression *in vitro* and *in vivo* during virus infection and correlate it with viral burden. *Heatr9* is induced by influenza virus and RSV. *Heatr9* knockdown during viral infection was shown to affect chemokine expression. Our studies identify *Heatr9* as a novel inflammatory and virus infection induced gene that can regulate the induction of specific cytokines.

## Introduction

Although one of the most well studied models of an acute immune response to an infectious pathogen, there are still many areas which remain to be elucidated in influenza virus infection. In the advent of new, more affordable technologies, like whole transcriptome RNA Sequencing [1, 2], RNA interference screens [3], CRISPR/Cas screenings [4, 5], global methylation profiling [6], Chromatin Immunoprecipitation (ChIP) assay [7, 8], and others, more knowledge is constantly being acquired as to what constitutes the host immune response to influenza virus infection. These new strategies not only instruct us as to what changes occur during infection but also allow the potential elucidation of the functional effects of these changes during viral infection.

We previously reported using RNA sequencing of *in vivo* influenza virus infected lung alveolar epithelial cells (AEC) and identified a number of genes upregulated in both directly infected AEC and bystander AEC isolated from infected mouse lungs [9]. Our previous data highlighted the differences caused by direct viral infection, essentially focusing on genes differentially regulated only in infected cells. Those genes upregulated by general inflammation within the lung in AEC were not detected in this comparison. One gene in the top upregulated genes within both infected and bystander AEC, *Heatr9*, was significantly upregulated but had no known associated function. *Heatr9*, also known in humans as *Chromosome 17 open reading frame 66* (*C17orf66*) and in mouse as *HEAT repeat containing protein 9* (*Heatr9*) (Gm11435) is named for its HEAT protein domain containing repeats present in its sequence [10].

We were not the first to identify *Heatr9* as previous studies have identified it in mouse models of *Staphylococcus aureus* infections, as well as in RNA sequencing data from various virus infections, both *in vitro* in human cell lines and *in vivo* in mouse infections [11, 12]. According to Ensembl, there are multiple splice variants in humans of *Heatr9* (ENSG00000270379) ranging from approximately 0.5 to 2.5 kilobases in length but only one transcript in mice [13]. Using a yeast two hybrid array, publicly available data suggest that *Heatr9* could interact with Hemk1, a methyltransferase family member [14]. This would suggest that *Heatr9* is a translated protein and not acting as an RNA species.

HEAT repeats are protein structural motifs of approximately 37–43 residues in length and occur in protein sequences with anywhere from three to more than twenty repeats [15]. The name HEAT is derived from four proteins which contain multiple HEAT repeats: *H*untingtin, *e*longation factor 3, protein phosphatase 2*A*, and mammalian *t*arget of rapamycin 1 [16]. As an example, the PR65/A subunit of protein phosphatase 2A contains the HEAT repeats which stack upon each other and thus places them in a larger family of proteins known as solenoids [15]. The HEAT repeat structurally consists of two amphiphilic alpha helices connected by an intra-unit loop [17]. The alpha helices have little primary sequence homology but the structure is highly conserved as the internal residues of the helices form a hydrophobic region and the external residues are hydrophilic [18]. The proteins that contain these HEAT repeats have been divided into three groups based upon the structure of the protein: Group I proteins contain only consecutive HEAT repeats, Group II proteins contain HEAT repeats but with intrinsically disordered regions, and Group III proteins contain HEAT repeats as well as another structural domain with a characterized function [18]. Although Group III proteins may have a known function due to the other distinct domain, the presence of HEAT repeats in general is associated with no single specific function, although many suggest that these repeats are in general domains for protein protein-interactions [19].

Here we report that during infection with influenza virus, *Heatr9* transcripts are upregulated in the lung, specifically within alveolar epithelial cells. Furthermore, in an *in vitro* model of influenza virus and respiratory syncytial virus (RSV) infection with A549 cells, *Heatr9* is highly upregulated after infection and can be induced, albeit to a reduced extent, by treatment with infected cell culture supernatants or exposure to a combination of inflammatory cytokines. *Heatr9* knockdown experiments in virus infected cell cultures suggest that it plays a role in cytokine expression of neighboring genes. These findings suggest that Heatr9 may be a novel inflammation responsive gene and plays an important role during influenza virus infection in cytokine expression, specifically chemokine ligands and thus regulating cell recruitment and inflammation in the lung.

## Materials and methods

### Animals and *in vivo* influenza virus infections

Mice were maintained in facilities according to the American Association for Accreditation of Laboratory Animal Care at Drexel University College of Medicine (Philadelphia, PA, United States of America) or the European Directive 2010/63EU at Erasmus MC University Medical Center (Rotterdam, The Netherlands). Animal protocols were prospectively approved and performed according to protocol 210208 (approved by the Institutional Animal Care and Use Committee (IACUC) at Drexel University College of Medicine) or work protocol 15-179-08 (approved under Project Proposal (AVD101002015179) by the animal welfare body (AWB) of the Instantie voor Dierwenwelzijn (IvD) at Erasmus MC University Medical Center). Animal experiments were conducted in compliance with United States government law under the Animal Welfare Act and the Public Health Service Policy on Humane Care and Use of Laboratory Animals or following the Netherlands’ government laws of the Centrale Commissie Dierproeven. C57BL/6 mice were obtained from The Jackson Laboratories or registered vendors of The Jackson Laboratories C57BL/6 mice (Charles River France for studies performed in the Netherlands). Adult (8–12 week old) female C57BL/6 mice were infected with 1.2 x 10^5^ PFU A/Puerto Rico/8/1934 (PR8) influenza virus in 40 microliters of sterile saline intranasally while anaesthetized with isoflurane or avertin (2,2,2-tribromoethyl alcohol). Mouse body weight was measured daily, with any mice losing greater than 25% of initial body weight being humanely euthanized. Mouse body weight was monitored until return to pre-infection weight was regained, a humane endpoint was reached, or the experimental endpoint was achieved. Humane endpoints include any mouse demonstrating greater than 25% loss of initial body weight, abnormal behavior, tumors, severe wounds from fighting, or prolonged sickly appearance (i.e. unkempt fur, reduced drinking, scruffy appearance, slowed locomotion) than would normally be expected. Mice were euthanized at designated time points and lungs were removed and stored in TRIreagent (MRC, Cincinnati, OH, TR-118 500) for further processing. Humane euthanization was performed with carbon dioxide gas (10% displacement of cage volume per minute)(Drexel University College of Medicine) or via cervical dislocation (Erasmus University Medical Center). Fifty-one mice were used during the course of this study. Three mice were removed from the study due to greater than expected weight loss amounts to approximately 5% removal.

### *In vitro* influenza virus infections

The green fluorescent protein expressing A/Puerto Rico/8/1934 (PR8-GFP) influenza virus was a generous gift of Dr. Adolfo Garcia-Sastre (Mount Sinai Hospital). A549 cells were purchased from ATCC (ATCC® CCL-185, Manassas, VA) and subcultured every 2–3 days while being maintained in DMEM media (Gibco, United Kingdom) containing 10% fetal bovine serum (FBS), 2 mM glutamine (Gibco), 200 units/mL penicillin, and 200 units/mL streptomycin (Gibco). Prior to infections, A549 cells were washed with serum-free DMEM (Gibco, United Kingdom) containing 2 mM glutamine (Gibco), 200 units/mL penicillin, and 200 units/mL streptomycin (Gibco). Virus containing serum-free media was then added to the cells with multiplicity of infection (MOI) of 1. Cells were incubated with influenza virus for 2 hours at 37°C in 5% CO_2_ before being washed with serum-free media to remove excess virus. Where plaque assays were performed, serum-free media was replaced; in all other instances 10% FBS media was added to the cells after infection and were returned to the incubator for the indicated time period.

### *In vitro* respiratory syncytial virus (RSV-A2) infections

A549 cells were trypsinized (25-052-CI, Corning, Manassas, VA), counted, and RSV virus (lab strain RSV-A2) at a ratio of 1 MOI (2.5 x 10^6^ TCID_50_ or approximately 1.7 x 10^6^ PFU) was added to cells in suspension for 1 hour. Cells were washed, plated at 1.0 x 10^6 cells per well in a 6 well plate and incubated for 48 hours at 37°C prior to isolation of RNA.

### cDNA synthesis and quantitative real time PCR

cDNA was generated from isolated RNA using the Applied Biosystems High Capacity cDNA Synthesis kit (Lithuania). Quantitative real time PCR’s to determine gene expression were performed using Taqman Universal master mix as well as well as Taqman gene expression assays for *Gapdh* (Assay ID: Hs02758991_g1 for human, Mm99999915_g1 for mouse), *Ccl5* (Assay ID: Hs00982282_m1), *Ccl4* (Assay ID: Hs99999148_m1), *Mmp28* (Assay ID: Hs00425232_g1), C17orf66/Heatr9 (Assay ID: Hs00330469_m1 for human, Mm03015844_m1 for mouse), *Ifnl1* (Assay ID: Hs00601677_g1), *Ifna2* (Assay ID: Hs00265051_s1), *Ifnb1* (Assay ID: Hs01077958_s1), *Ifnl2* (Hs00820125_g1), *Isg15* (Assay ID: Hs01921425_s1), and *Oas1* (Hs00973637_m1) (for primers, see S1 Table). For viral load quantification, equivalent mRNA amounts (1500 ng) were used as input in a cDNA reaction with specific primers to amplify influenza MP protein (Sequence: TCTAACCGAGGTCGAAACGTA). Synthesized cDNA was used in a standard qRT-PCR reaction with specific primers to amplify influenza MP cDNA (sense: AAGACCAATCCTGTCACCTCTGA and antisense: CAAAGCGTCTACGCTGCAGTCC) with an influenza-specific MP protein targeting probe (TTTGTGTRTCACGCTCACCGT). Viral units were quantified by standard curve to approximate PFU values from the initial virus inoculum. Total viral load was then back calculated based upon input RNA and total lung mass. Fold induction of gene expression was calculated based upon untreated or uninfected control samples using the delta Ct method. For those Ct values where the target was undetermined after 40 cycles, or had a Ct value above 35, a Ct value of 35 was used for calculation purposes. For detection of *Heatr9* in uninfected mouse organs, tissues were collected and processed as described above for isolation of RNA, generation of cDNA, and analysis by qRT-PCR. Following qRT-PCR, products were electrophoresed on a 2.5% agarose gel at 100 volts for approximately 60 minutes before being visualized using a Bio-Rad Gel Doc XR+ station.

### Cytokine treatment

IFN-β1 (300-02BC), TNF-α (AF-300-01A), and IL-1β (AF-200-01B) were purchased from Peprotech (Rocky Hill, NJ). Lyophilized cytokines were resuspended in RPMI (Gibco) supplemented with 10% FBS, 2 mM glutamine (Gibco), 100 units/mL penicillin, and 100 units/mL streptomycin (Gibco) at a concentration of 10 μg/mL. IFN-λ (IL-29) (1589-IL-025) was purchased from R&D Systems (United States of America) and resuspended in sterile Hanks Buffered Saline Solution (HBSS). Cytokines were used at a concentration of 10 ng/mL and incubated with A549 cells for 16 hours prior to washing with HBSS and cell resuspension in TRIreagent (MRC, Cincinnati, OH, TR-118 500). Samples were frozen at -80 degrees Celsius prior to RNA isolation.

### CCL5 ELISA

Infected A549 cell culture supernatants were collected and frozen until processing. Supernatants were thawed and diluted for quantification using the Human CCL5/RANTES Quantikine ELISA kit (R&D Systems, Minneapolis, MN, DRN00B). Manufacturer’s instructions were followed and plate O.D. was read by a Versa Max tunable microplate reader.

### Gapmer transfections

Lyophilized gapmers were resuspended in nuclease free water (10336503, Fisher Scientific, Belgium,) at a concentration of 50 μM, aliquoted, and frozen at -80° Celsius. Gapmers were designed and under proprietary protection from Qiagen. Catalogue numbers for the gapmers used are LG00207464-DFB (Heatr9-targeting) and LG00000002-DFB (control gapmer). Transfection Media was prepared by supplementing RPMI media (Gibco) with 2 mM glutamine (Gibco), 100 units/ml penicillin (Gibco), 100 units/ml streptomycin (Gibco), and 1% 1M HEPES. Prior to transfection, gapmers were diluted to 200 nM in transfection media (solution A). Lipofectamine 2000 reagent 11668019, Life technologies, Carlsbad, CA) was diluted in transfection media (solution B). Solution A containing gapmers (final concentration 100 nM) was combined with Lipofectamine containing solution B at a 1:1 ratio. Cocktail of gapmers and lipofectamine containing transfection media was allowed to incubate for approximately 10 minutes at room temperature before being added dropwise to cells in a volume of 500 microliter per well of a 6 well plate.

### Statistical analysis

For statistical analysis, data were subjected to normality test prior to conducting statistical tests in order to determine whether a parametric (normally distributed data) or nonparametric (non-normally distributed data) tests should be performed. All computational analysis was performed using GraphPad Prism version 5.

### Database

Gene expression omnibus (GEO): GSE119123

## Results

### *Heatr9* is upregulated during influenza virus infection *in vivo* in mice

When we analyzed the by RNA-seq the transcriptome of *in vivo* infected AEC and compared sorted AEC from influenza virus infected C57BL/6 mice with uninfected AEC, *Heatr9* was a transcript found to be more abundant in both bystander AEC as well as directly infected AEC at three days post infection (data from [9]). Compared to uninfected AEC, *Heatr9* was upregulated 481-fold in directly infected AEC (S2A Table) and 203-fold in bystander AEC (S2B Table) [9] (data accessible from Gene Expression Omnibus: GSE119123). In order to understand the general expression pattern and to determine whether this was a gene that is induced during inflammatory circumstances, we performed qRT-PCR to determine its expression in uninfected mouse organs, including spleen, lung, liver, and kidneys (S1 Fig) (S3 Table). We found evidence of *Heatr9* transcripts in all organs tested at the steady state.

**Fig 1:**
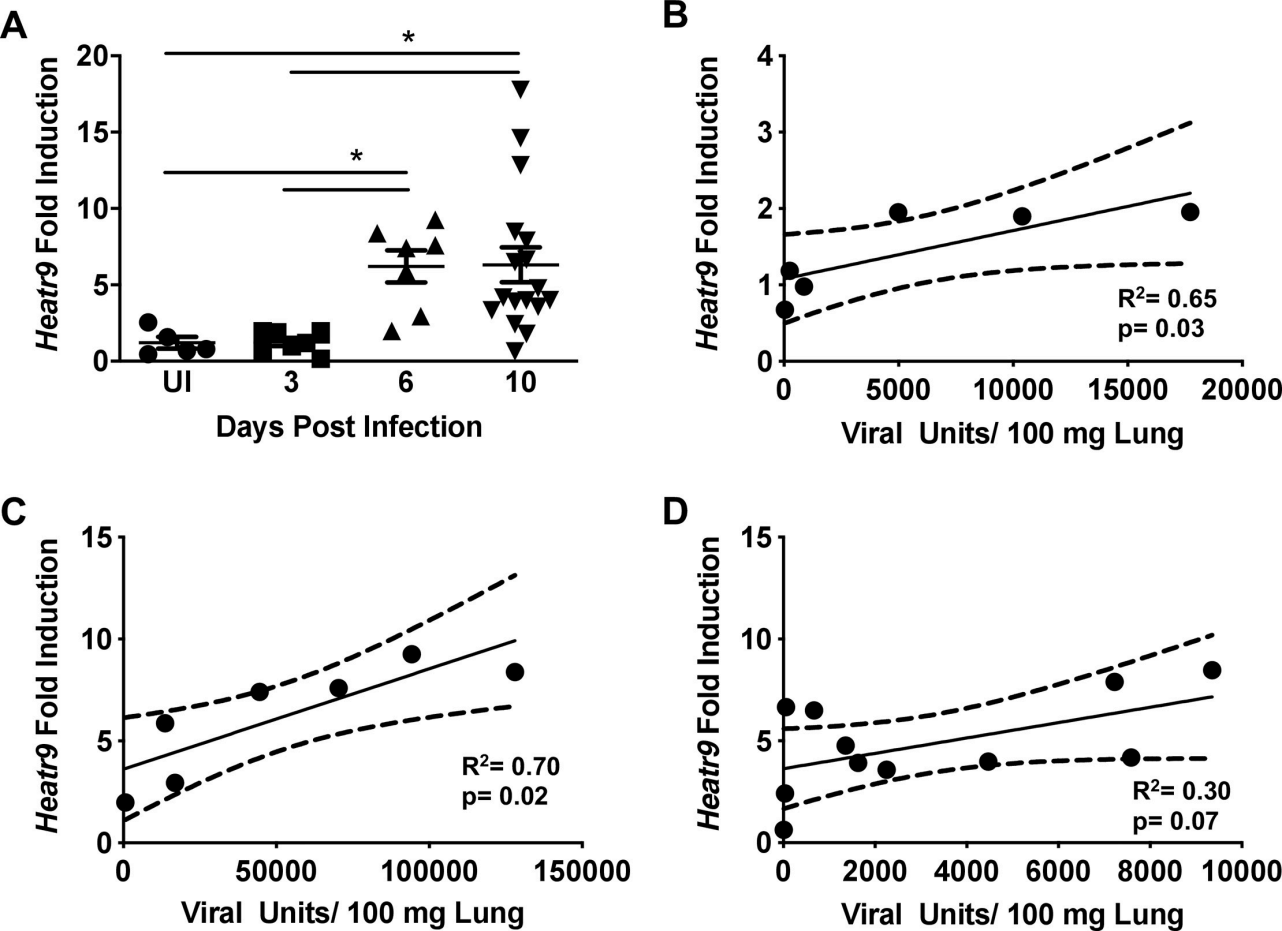
Viral load and *Heatr9* expression during acute influenza virus infection in C57BL/6J mice. Pooled experimental data of *Heatr9* expression was tracked over the course of acute influenza virus infection from RNA isolated from PR8 influenza virus infected C57BL/6 mouse lungs (A). The expression of *Heatr9* from individual experiments (determined by fold induction compared to uninfected control mouse lungs) was correlated with viral titers at days 3 (B), 6 (C), and 10 (D) post infection. Each dot represents a single mouse. Results representative of 2 or more independent experiments. Viral Units were determined by qRT-PCR based method in which the MP viral transcript was detected from cDNA synthesized from isolated mouse lung RNA. For statistical analysis, (A) a nonparametric One-way ANOVA was performed (Kruskal-Wallis test) with a post hoc Dunn’s multiple comparison test to determine significance (* indicates p<0.05). For correlations (B, C, and D), a Spearman correlation test for significance was performed. Significant correlations are considered those with p<0.05. Each dot represents an individual mouse. Due to insufficient material, the correlation between Heatr9 induction and viral burden could not be performed for all mice. This is the reason why more data points are present in 1A compared to 1D.

We then wanted to confirm the induction of *Heatr9* during influenza virus infection by qRT-PCR from infected mouse lungs and to determine the kinetic of expression during acute virus infection. In order to do so, we infected C57BL/6 mice intranasally with A/Puerto Rico/8/1934 influenza virus and harvested lungs at days three, six, and ten post infection to determine viral burden (S2 Fig) and compared the expression of *Heatr9* in whole infected lungs across all time points to uninfected control lung RNA. *Heatr9* was found to be induced by influenza virus infection plateaued at day six where its expression remained constant until day ten (Fig 1A). As these were whole lung homogenates, the RNA accounts for not only AEC but other cell types, including immune cells, many of which have already infiltrated the lung by day three post infection. Thus, it is more than likely that the level of *Heatr9* expression in AEC is underestimated in these whole lung homogenates. Due to variability in viral burden, we correlated the viral titer with the induction of *Heatr9* expression during the course of influenza virus infection. Viral units were determined by qRT-PCR analysis from RNA isolated from lung homogenates where the Ct values of detectable MP transcripts were equated to a standard curve of known plaque forming unit equivalents. In Fig 1B–1D, it is apparent that at day three (Fig 1B) and day six post infection (Fig 1C), the induction of Heatr9 is positive and significantly correlative with the viral burden. On day ten (Fig 1D) this trend is less apparent, however this may be a result of more variability as lung homogenates become undetectable for viral transcripts, demonstrating the recovery and clearing of the infection. This suggests a graded expression of *Heatr9* depending upon viral burden, indicating that greater viral transcripts stimulate greater *Heatr9* induction and further support the idea that viral infection drives *Heatr9* expression. We thus, have confirmed *Heatr9* transcript induction during acute influenza virus infection *in vivo*.

#### *Heatr9* is upregulated during direct viral infection *in vitro* and is cytokine inducible

**Fig 2:**
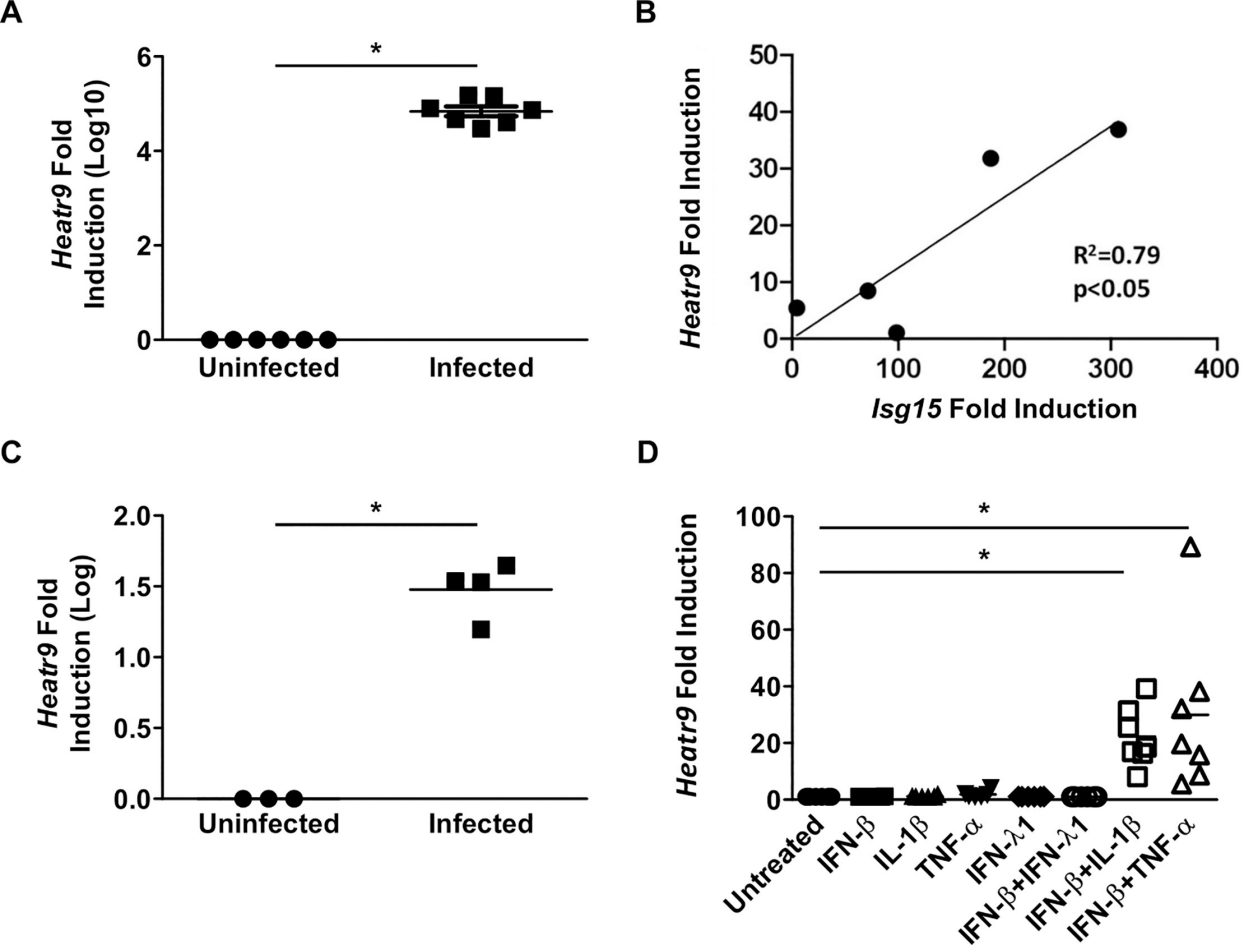
*Heatr9* is induced upon viral infection and treatment with cytokines. A549 human lung cells were infected with 1 MOI of PR8 influenza virus and incubated for 24 hours before RNA was isolated to assay for *Heatr9* expression by qRT-PCR (A). A549 cells were infected with RSV-A2 and RNA was isolated at 48 hours post infection. *Heatr9* induction was plotted against *Isg15* induction (B). A549 cells were infected with 1 MOI of PR8 and incubated for 24 hours prior to removal of culture supernatant, which was then incubated with uninfected A549 cells. RNA was isolated from uninfected A549 incubated with supernatant of infected A549 cells for 24 hours prior to RNA isolation for assaying for *Heatr9* induction (C). A549 cells were incubated with cytokine(s) for 16–24 hours in the absence of infection and *Heatr9* expression induction was assayed by qRT-PCR (D). For statistical analysis, unpaired Student’s t test (A, C), linear regression analysis (B), and One-way ANOVA with a post hoc Tukey multiple comparisons (D) were performed. Asterisk (*) indicates a p<0.05.

Having confirmed the induction of *Heatr9* expression *in vivo* during influenza virus infection in the lungs of mice, we wanted to confirm *Heatr9* upregulation following virus infection *in vitro*. Using a relevant human lung adenocarcinoma cell line, A549 cells, we quantified the induction of *Heatr9* at 24 hours post infection with 1 MOI A/Puerto Rico/8/1934-GFP. Following influenza virus infection *Heatr9* is highly upregulated more than 1000-fold over uninfected cells (Fig 2A). From qRT-PCR analysis, it appears that *Heatr9* is nearly undetectable in uninfected cells (Ct values by qRT-PCR >35 cycles). In order to determine whether *Heatr9* induction is specific for influenza virus or can be upregulated by other respiratory viruses, we infected A549 cells with respiratory syncytial virus (RSV) lab strain A2 (RSV-A2). Infection of A549 cells with 1 MOI of RSV-A2 induced *Heatr9* expression with a significant positive correlation when plotted against interferon stimulated gene 15 (*Isg15*) induction (Fig 2B). This suggested that *Heatr9* induction is part of a response to viral infection.

As bystander mouse AEC also upregulated *Heatr9 in vivo*, we then attempted to induce *Heatr9* expression by treating uninfected A549 cells with culture supernatants from influenza virus infected A549 cells. This was meant to effectively mimic, the exposure to inflammatory signals in the absence of infection. As seen in Fig 2C, transfer of infected culture supernatant from PR8-GFP infected A549 culture supernatants at 24 hours post infection was capable of inducing *Heatr9* expression, albeit not as strongly as direct virus infection. Although it could be suggested that infectious virus produced in the supernatant of these infected cultures could infect these uninfected cells when transferred, we replaced the media with serum containing media after infection to inhibit newly released virions from infecting other cells although it cannot be ruled out that receptors on the surface could also be sensing influenza virus in the absence of direct infection. As *Heatr9* appeared to be induced in the absence of direct infection, we attempted to identify if any cytokines were capable of inducing *Heatr9* expression in the absence of infectious virus. Single cytokine treatment was insufficient to induce *Heatr9* expression, however treatment with interferon-beta 1 (IFN-β1) in combination with either interleukin-1 beta (IL-1β) or tumor necrosis factor alpha (TNF-α) was capable of inducing *Heatr9* expression to a similar extent as transfer of infected A549 cell culture supernatant (Fig 2D). This induction however was still much lower than that observed with direct infection of A549 cells.

#### The location of *Heatr9* in the genome

In order to understand how *Heatr9* is regulated and its potential function, we examined its location in the human and mouse genomes. Interestingly, *Heatr9*, in both mice and humans, is located immediately upstream to *Ccl5* (S3 Fig), a gene also among the most upregulated in both infected and bystander AEC (data from Hancock et al. [9]; data deposited in Gene expression omnibus accession number [GSE119123]). Further surveying of the genome revealed that between the end of *Ccl5* and beginning of *Heatr9* coding loci, there were no predicted promoter regions [13]. Being that both of these transcripts are in close proximity and were both highly upregulated, this suggested a relationship between these two genes’ transcriptional expression with a possible common transcriptional regulation.

#### *Heatr9* knockdown reveals a putative connection between *Heatr9* and an effect on cytokine expression

**Fig 3:**
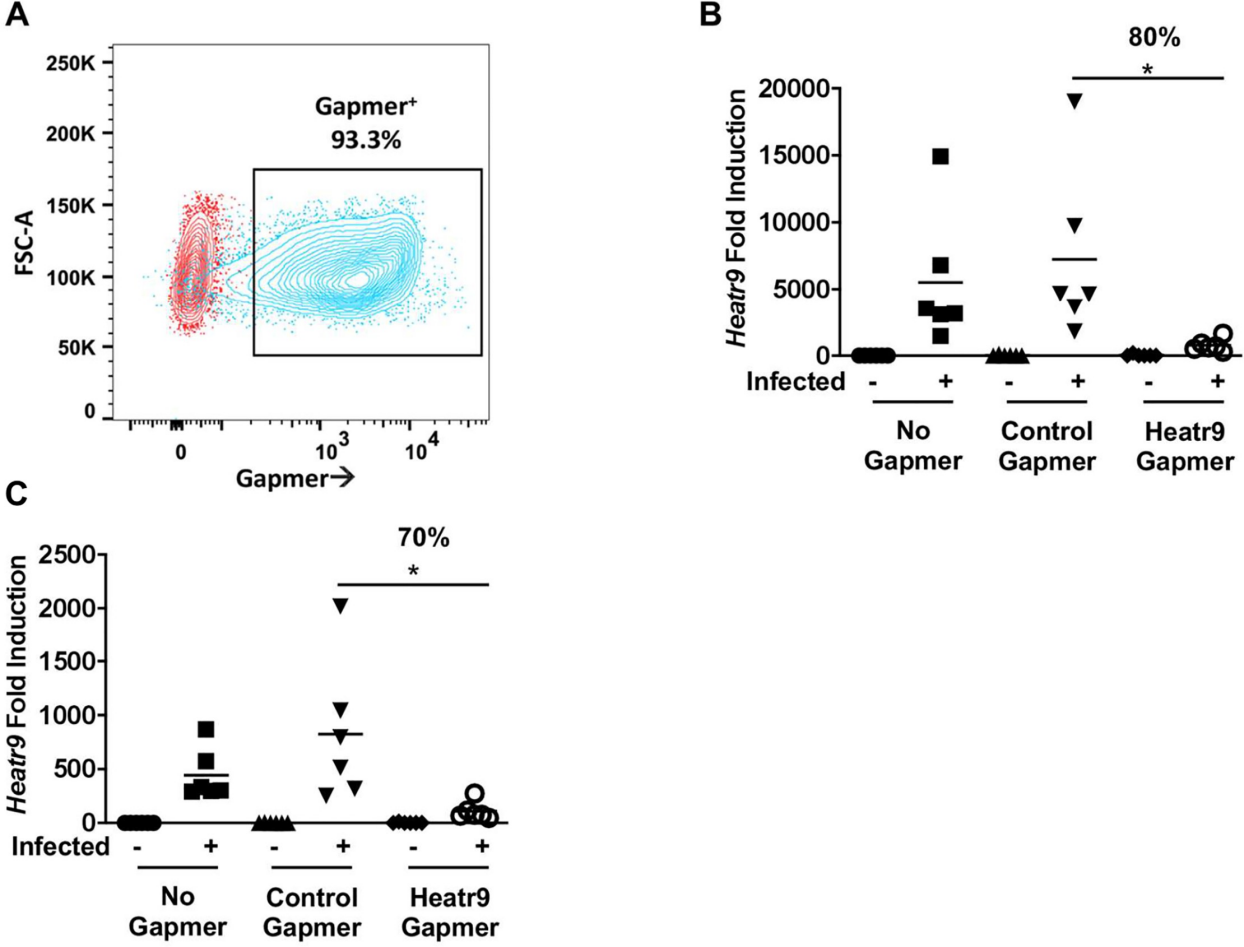
Gapmer-mediated knockdown of *Heatr9*. A549 cells were left untreated (No Gapmer), transfected with gapmers encoding no targeting sequence (Control Gapmer), or gapmers targeting *Heatr9* (Heatr9 Gapmer). Transfection efficiency was determined by the frequency of FAM positivity by flow cytometry (A). Dot plots show an overlay of both the untreated population (red) and Heatr9 gapmer transfected population (blue). Transfection in infected cell cultures corresponded to approximately 80% knockdown of *Heatr9* at 24 hours post transfection (B) and 70% at 48 hours post transfection compared to control infected and transfected cell cultures (C). Each dot represents an independent experiment. For statistical analysis, Student’s t test was performed to compare control gapmer and Heatr9 targeting gapmer transfected cell cultures. Asterisk (*) indicates a p≤0.05.

To study the function of *Heatr9* during influenza virus infection, we knocked down *Heatr9* expression via transfection of gene-targeting gapmers into A549 cells followed by infection. Gapmers (commercially available and custom designed) are transfectable locked nucleic acid (LNA) flanking molecules with a central DNA sequence region that contains gene-targeting homology to its RNA complement (thereby conferring specificity). Upon binding to the target RNA, degradation occurs by cellular RNAses and use of a 6-carboxyfluorescein (FAM)-tag allows for quantification of transfection efficiency. Thus efficient transfection into A549 cells was detectable via FAM positivity determined by flow cytometry which revealed a greater than 90% transfected population (Fig 3A). Using gapmer-mediated knockdown of *Heatr9* in A549 cells during influenza virus infection, we were able to reduce *Heatr9* levels by greater than 80% at 24 hours post infection (48 hours post transfection) (Fig 3B) and by more than 70% at 48 hours post infection (Fig 3C) (72 hours post transfection) compared to the respective time point control gamer transfected influenza virus infected A549 cells.

**Fig 4:**
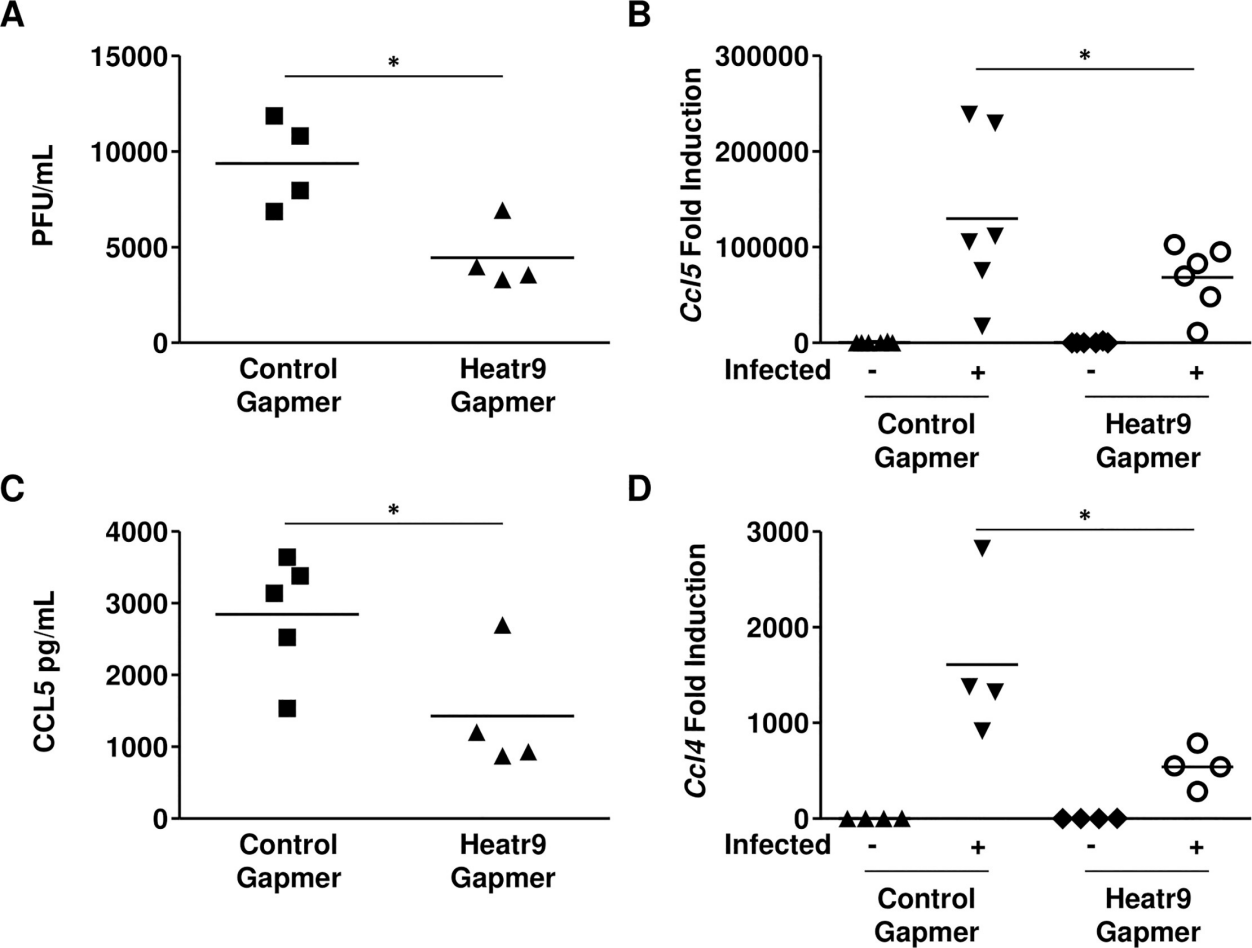
*Heatr9* knockdown affects viral production and chemokine induction. *Heatr9* gapmer transfected cell cultures produced less infectious virus in the culture supernatants as determined by MDCK plaque assay compared to non-targeting control gapmer transfected cell cultures (A). Knockdown of *Heatr9* resulted in less induction of *Ccl5* at the transcript level (B) as determined by qRT-PCR resulting in less protein (C) in infected culture supernatants. *Heatr9* knockdown also resulted in less *Ccl4* induction (D). Each dot represents an independent experiment. Lines between data points indicate matching cells from the same experiment. All cells were exposed to the transfection reagent. Non-targeting gapmer indicates transfection with a control gapmer with no specific target (Control Gapmer); Heatr9 gapmer indicates transfection with a *Heatr9* targeting specific gapmer (Heatr9 Gapmer). For statistical analysis, paired Student’s t tests were performed. Asterisk (*) indicates a p≤0.05. No indication signifies a nonsignificant result.

*Heatr9* knockdown resulted in a number of changes in A549 cell responses to influenza virus infection. *Heatr9* knockdown A549 cultures produced significantly although modest less infectious virions at 24 hours as compared to control gapmer transfected A549 cultures (Fig 4A). Because of *Heatr9* and *Ccl5*’s close proximity in the genome and the possibility that they could be transcriptionally co-regulated, we assayed for differences in *Ccl5* transcription induction in *Heatr9* gapmer transfected A549 cultures. Interestingly, we found a significant reduction in the expression of *Ccl5* at the transcriptional level in infected *Heatr9* knockdown A549 cell cultures (Fig 4B). In order to understand if this difference in induction resulted in reduced secretion of CCL5 at the protein level, we quantified CCL5 in the supernatants of infected cultures. We detected significantly reduced CCL5 protein in *Heatr9* gapmer transfected cell culture supernatants compared to cell culture supernatants transfected with control gapmers (Fig 4C). We also investigated whether knockdown of *Heatr9* could impact the expression of closely associated transcript *Ccl4* which was also found to be upregulated after influenza virus infection in alveolar epithelial cells (data from Hancock et al. [9]). This transcript also lies in close proximity (further upstream of *Heatr9*) in both human and mouse genomes (S3 Fig). This could possibly indicate that *Heatr9* may play a role in chemokine induction of genes lying in cross proximity to it within the genome. We found that *Ccl4* gene expression was also significantly reduced in *Heatr9* gapmer knockdown A549 cells (Fig 4D).

**Fig 5:**
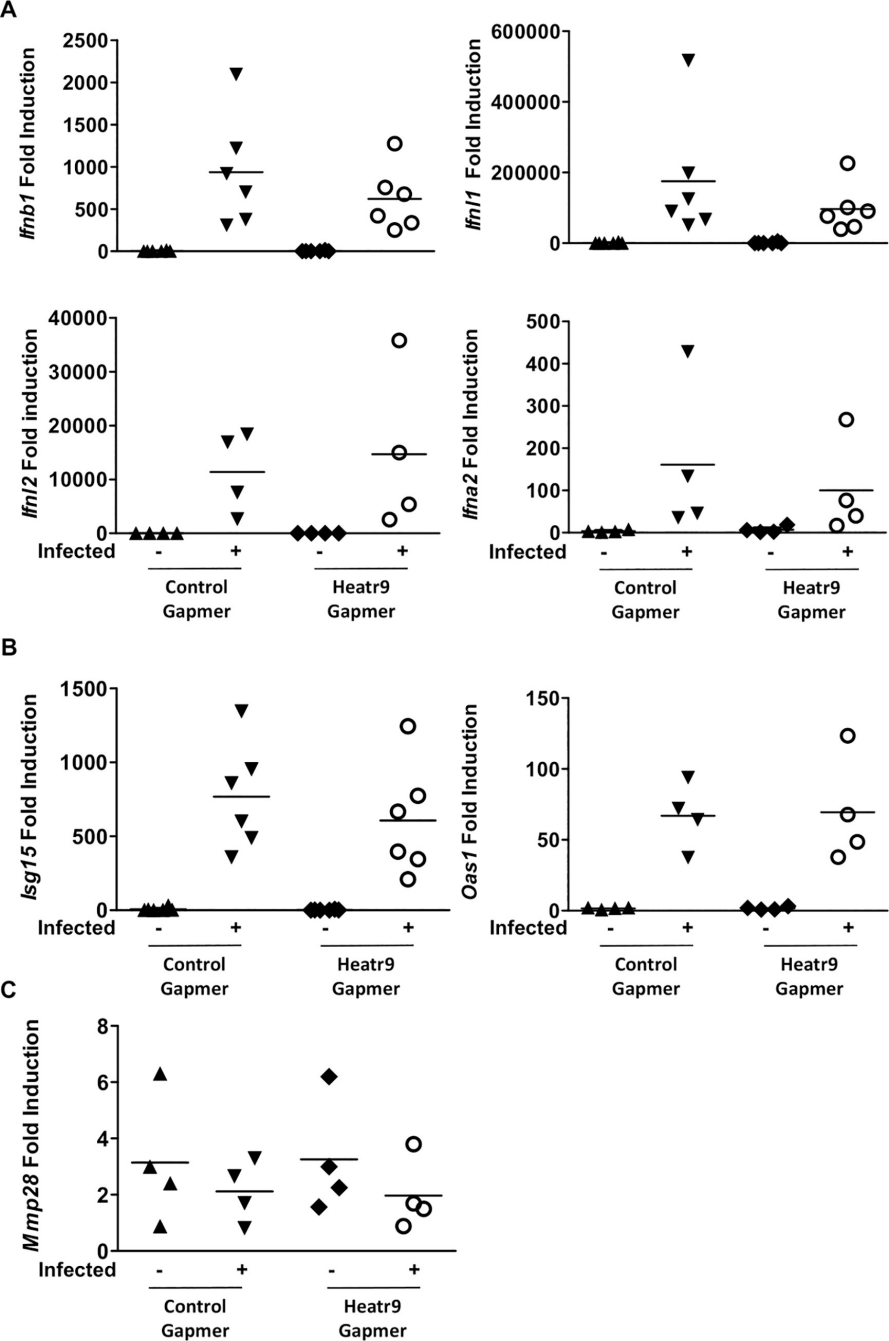
*Heatr9* gapmer transfection does not affect gene induction of interferons or all genes in proximity to *Heatr9*. Heatr9 knockdown does not affect *Ifnb1*, *Ifnl1*, *Ifnl2*, or *Ifna2* gene induction (A) or induction of interferon stimulated genes *Isg15* or *Oas1* (B). Genes near the Heatr9 loci and also found to be upregulated during influenza virus infection were also unaffected by *Heatr9* knockdown, as determined by *Mmp28* transcript induction (C). Each dot represents an independent experiment. Lines between data points indicate matched experiments. For statistical analysis, paired Student’s t tests were performed. No indication signifies a nonsignificant result.

In order to understand if this difference in induction in *Ccl5* and *Ccl4* was specific for these chemokines or whether knockdown of *Heatr9* had a global effect on cytokine expression, we assayed for induction of type I IFN, IFN-β1 and IFN-α2 (*Ifnb1* and *Ifna2*), and type III IFN, IFN lambda 1 and 2, (*Ifnl1* and *Ifnl2*) as well as interferon stimulated genes, IFN stimulated gene 15 and 2’5’-oligoadenylate synthetase 1 (*Isg15* and *Oas1*, respectively). Analysis of gene expression showed no significant differences in the induction of *Ifnb1*, *Ifna2*, *Ifnl1*, *Ifnl2*, *Isg15*, or *Oas1* after influenza virus infection in *Heatr9* gapmer transfected cell cultures (Fig 5A and 5B). In addition, another gene in close proximity to *Heatr9* that was found to be upregulated in epithelial cells, *Mmp28*, was also unaffected by *Heatr9* knockdown (Fig 5C) [9]. This suggests that knockdown of *Heatr9* has a specific effect on chemokine induction, and not a broad effect on cytokines or genes located in the proximity of the *Heatr9* loci. This suggested a specific effect of Heatr9 knockdown on *Ccl5* and *Ccl4* induction but not on *Mmp28*, which were all found to be upregulated in AEC after influenza virus infection and lie in close proximity to *Heatr9* in the genome.

## Discussion

*Heatr9* is a newly identified transcript that was found to be highly upregulated during influenza virus infection in AEC isolated from day three PR8-GFP influenza virus infected mice by RNA-Seq. The upregulation of this gene was further confirmed by *in vitro* assays of viral infection. We simplified the system to isolate the effect of direct infection on epithelial cells from effects of other cell types present in infected lungs by examining *Heatr9* in cell lines. An *in vitro* model of influenza virus infection was utilized with A549 cells, a human lung epithelial cell line, effectively removing the effect of any infiltrating immune cells or cytokines produced by these cells. In this model, we were able to show that direct viral infection of A549 cells with either influenza virus or RSV-A2 was capable of inducing *Heatr9* expression. As the bystander cells in the RNA-Seq data suggested, *Heatr9* could be upregulated in the absence of direct viral infection. Use of A549 cells showed that transfer of infected culture supernatant or cytokines used in combination were capable of inducing *Heatr9* expression, albeit not to the same extent as direct viral infection. Thus the intracellular recognition of viral products is the most potent inducer of *Heatr9* expression. HEAT repeats are conserved at the tertiary structure as two amphiphilic helices connected by an intra-unit loop [18]. The presence of this domain does not give a strong indication as to what the function of *Heatr9* is during infection as HEAT repeats can be found in a number of different proteins [18]. However, it is interesting to speculate that perhaps Heatr9 protein could act as a checkpoint for chemokine expression, as knockdown of *Heatr9* induces a subsequent reduction in specific chemokine genes. It remains possible that low grade inflammatory stimuli are insufficient in inducing *Heatr9* to appreciable levels which is necessary for augmented expression of chemokines and that during active viral infection, *Heatr9* protein increases the expression of chemokines to allow further recruitment of immune cells. This would fit with its undetectable expression in uninfected status in epithelial cells, and its rapid induction and effect on *Ccl5* and *Ccl4* transcripts when expression was inhibited. Moreover, HEAT repeats are known to be found in proteins associated with the DNA structure and transcriptional control [19]. Thus *Heatr9* has the potential to act at the DNA level regulating the transcription of chemokines; this is supported by the finding that *Heatr9* knockdown reduced *Ccl5* and *Ccl4* mRNA transcripts. Leukocyte recruitment during influenza virus infection must be carefully controlled as increased immune cell recruitment could have deleterious effects for the host and increase immunopathogenesis [20]. However, it cannot be ruled out that *Heatr9* may act as lncRNA, although mice only have one transcript, humans have multiple splice variants, some of which are not translated [13]. In this way *Heatr9* might act as a possible co-activator or stabilizing lncRNA in transcriptional complexes that drive chemokine expression. *Heatr9* deficiency then would theoretically result in poor cell recruitment and most likely affect pathogen clearance.

Previous data on single nucleotide polymorphisms (SNPs) associated with *Ccl5* and *Heatr9* also support the association of these genes. One study identified four *Ccl5* gene SNPs that were associated with elevated albumin excretion in non-diabetic patients, which is a prognostic indicator of vascular damage and could lead to nephropathy, a pathological phenomenon driven by CCL5 inflammation [21]. Although none of the four SNPs found to affect *Ccl5* were found in the *Heatr9* gene itself, a study performed to identify SNPs affecting gene expression listed all four of the SNPs to be associated with *Heatr9* expression [22]. Another study also focused on SNPs in *Ccl5* that were associated with reduced serum levels of this chemokine, as a correlate of protection for type 1 diabetes [23]. The three SNPs listed in this study were also found to be associated with *Heatr9* expression and furthermore, one SNP, rs2306630, is located within the Heatr9 gene itself [22, 23]. These human studies further support the connection between *Heatr9* and *Ccl5* expression levels and a possible contingent relation whereby when *Heatr9* is affected, *Ccl5* expression is also affected.

The induction of *Heatr9* in infected cells suggests that it may play a role either in host defense or it is hijacked by the virus for its own advantage. One way to reconcile these two contrasting roles is the possibility that Heatr9 could be important for transcriptional pathways. Our studies suggest that downregulation of Heatr9 affects CCL5 proteins levels as well as virus production. As other HEAT repeat domain containing proteins have already been implicated in transcriptional control and chromatin architecture, and influenza virus replicates within the nucleus, it remains possible that it is necessary for gene transcription of both the host response and virus production [16, 19]. Very little is known about *Heatr9*’s expression and potential function in cells. Apart from some expression data in alveolar macrophages [12] other public databases find that *Heatr9* is expressed within the lymph node, lung, brain, adrenal gland, intestines, spleen, and small intestines in humans and the brain, spleen, lung, testis, thymus, and intestines in mice [14]. This expression pattern is in agreement with our own survey of mouse tissues for *Heatr9* expression (S1 Fig).

The studies presented here focus on epithelial cell expression of *Ccl5* and *Ccl4*, however other cell types express these chemokine ligands and further studies are required to assess whether those cells would exhibit a similar phenotype of reduced chemokine expression when *Heatr9* expression is reduced. Furthermore, our finding that *Heatr9* is present in multiple organs by qRT-PCR suggests that it is expressed quite ubiquitously. As chemokines are also expressed in many tissues in uninfected status, *Heatr9* could play a role in low level homeostatic chemokine induction in the absence of viral infection. Its cytokine induction and its regulation of chemokine expression also raise the question whether *Heatr9* is regulated in inflammatory and autoimmune diseases. Further studies *in vivo* and *in vitro* are required to elucidate the role *Heatr9* plays during respiratory virus infections, its molecular mechanism of action, and whether this transcript is upregulated in other inflammatory conditions or whether it is lung and respiratory tract-specific.

## Supporting information

S1 Fig Heatr9 expression in selected mouse tissues. The expression of Heatr9 in mouse organs was determined by isolation of total RNA from the indicated tissues, cDNA was synthesized, and qRT-PCR was performed to detect Heatr9 expression where the probe spans exons. To confirm qRT-PCR results, products of reaction were visualized by gel electrophoresis. Image shows the presence of bands at the expected amplicon size for the gene expression assay used (63 bps). Products were electrophoresed on an agarose gel to visual amplicons. Two housekeeping genes (*Gapdh* at 109 bps and *B2m* at 77 bps) were used as controls. Lowest band on the ladder indicates 100 bps (indicated with arrow).
(DOCX)

S2 Fig Viral burden during influenza virus infection in mice. C57BL/6 mice were infected intranasally with influenza A virus A/Puerto Rico/8/1934. At time points indicated, animals were euthanized and a portion of the lungs was used to determine viral burden based upon viral transcripts detected in RNA isolated from lung homogenates and normalized per milligram of tissue.
(DOCX)

S3 Fig Genomic loci of *Heatr9* in humans and mice. *Heatr9* is located on chromosome 17 in humans (upper) and chromosome 11 in mice (lower). Figures show the genes flanking *Heatr9* including *Mmp28*, *Ccl5*, and *Ccl4*. Arrowhead direction indicates direction of transcription.
(DOCX)

S1 Table Ct values of Heatr9 expression in mouse tissues. Indicated mouse organs were from wildtype mice were stored in TRIZol and RNA isolated to perform cDNA synthesis and subsequent qRT-PCR analysis to detect Heatr9 expression in an uninfected C57BL/6 mouse. Table indicates the Ct values obtained from the qRT-PCR analysis and Fig 1 confirms the generation of the gene specific amplicon for the assay utilized by gel electrophoresis. The abbreviation “NTC” represents the “no template control” in which no genomic DNA was added.
(DOCX)

S2 Table A. List of Top 10 most upregulated genes in GFP+ AEC vs. uninfected AEC comparison. Heatr9 is the third most upregulated gene in the comparison of GFP+ AEC isolated from day 3 GFP-expressing- A/Puerto Rico/8/1934 infected mice versus uninfected AEC isolated from naïve mice. Asterisk (*) to demarcate the base mean as a normalized measure of averaged reads from all samples. B. List of Top 10 most upregulated genes in GFP- AEC vs. uninfected AEC comparison. Heatr9 is the fifth most upregulated gene in the comparison of GFP-AEC isolated from day 3 GFP-expressing- A/Puerto Rico/8/1934 infected mice versus uninfected AEC isolated from naïve mice. Asterisk (*) to demarcate the base mean as a normalized measure of averaged reads from all samples.
(DOCX)

S3 Table qRT-PCR Taqman gene expression assays. In order to accurately assess gene expression in mouse and human samples, validated Taqman Gene Expression Assays were utilized from Applied Biosystems. The assay Identifier indicates which pair of primer probes were used and specifications of the specific assays.
(DOCX)

S1 Raw Data (DOCX)
