# Supplementary material for: Heatr9 is an infection responsive gene that affects cytokine production in alveolar epithelial cells
Source: PLoS One. 2020 Jul 17;15(7):e0236195. doi: 10.1371/journal.pone.0236195 (PMC7367486; doi:10.1371/journal.pone.0236195)
Supplement: S1 Fig — The expression of Heatr9 in mouse organs was determined by isolation of total RNA from the indicated tissues, cDNA was synthesized, and qRT-PCR was performed to detect Heatr9 expression where the probe spans exons. To confirm qRT-PCR results, products of reaction were visualized by gel electrophoresis. Image shows the presence of bands at the expected amplicon size for the gene expression assay used (63 bps). Products were electrophoresed on an agarose gel to visual amplicons. Two housekeeping genes (Gapdh at 109 bps and B2m at 77 bps) were used as controls. Lowest band on the ladder indicates 100 bps (indicated with arrow). (DOCX) [file pone.0236195.s001.docx]

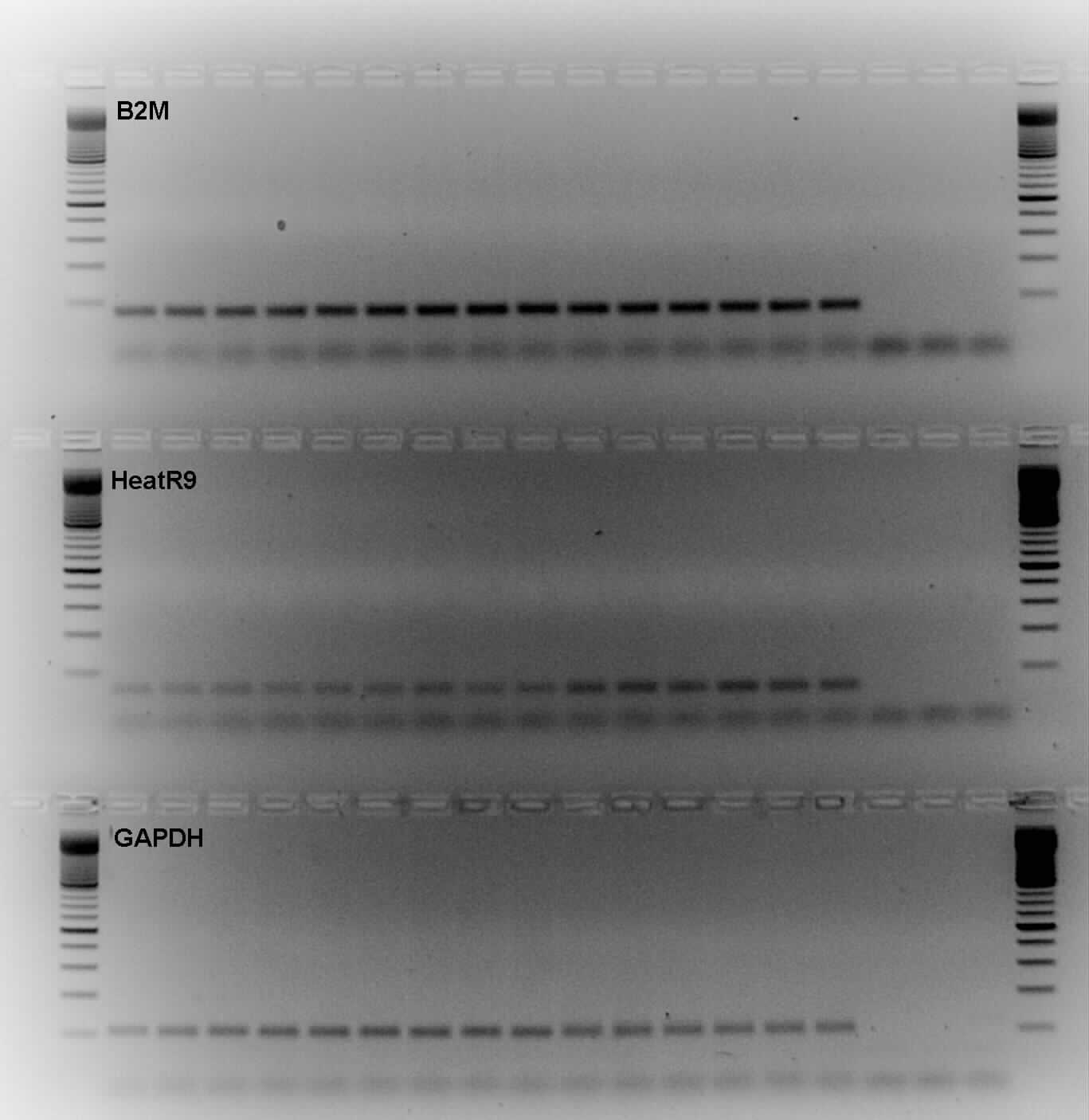


**Supplementary Figure 1**

**Heart**

**Spleen**

**Liver**

**Kidney**

**Negative**

**Lung**

**Heart**

**Kidney**

**Liver**

**Lung**

**Spleen**

**Negative**

**Heart**

**Kidney**

**Liver**

**Lung**

**Spleen**

**Negative**
