# Supplementary material for: Heatr9 is an infection responsive gene that affects cytokine production in alveolar epithelial cells
Source: PLoS One. 2020 Jul 17;15(7):e0236195. doi: 10.1371/journal.pone.0236195 (PMC7367486; doi:10.1371/journal.pone.0236195)
Supplement: S3 Fig — Heatr9 is located on chromosome 17 in humans (upper) and chromosome 11 in mice (lower). Figures show the genes flanking Heatr9 including Mmp28, Ccl5, and Ccl4. Arrowhead direction indicates direction of transcription. (DOCX) [file pone.0236195.s003.docx]

**Human Genome (Chromosome 17)**

***< Ccl3***

***< Ccl23***

***< Mmp28***

***< Ccl5***

***Taf15 >***

***Ccl4 >***

***< Ccl18***

***< Ccl14***

***< Tbc1d3b***

***< Ccl23***

***< Ccl15***

***< Ccl16***

***< Heatr9***

**Mouse Genome (Chromosome 11)**

***< Ccl5***

***< Mmp28***

***Wfdc18 >***

***< Ccl4***

***Taf15 >***

***< Heatr9***

***< Ccl3***

***Heatr6 >***

***Wfdc21 >***

***Wfdc17 >***

***< Ccl6***

***< Ccl9***

**Supplementary Figure 3**
