# Supplementary material for: Heatr9 is an infection responsive gene that affects cytokine production in alveolar epithelial cells
Source: PLoS One. 2020 Jul 17;15(7):e0236195. doi: 10.1371/journal.pone.0236195 (PMC7367486; doi:10.1371/journal.pone.0236195)
Supplement: S1 Table — Indicated mouse organs were from wildtype mice were stored in TRIZol and RNA isolated to perform cDNA synthesis and subsequent qRT-PCR analysis to detect Heatr9 expression in an uninfected C57BL/6 mouse. Table indicates the Ct values obtained from the qRT-PCR analysis and Fig 1 confirms the generation of the gene specific amplicon for the assay utilized by gel electrophoresis. The abbreviation “NTC” represents the “no template control” in which no genomic DNA was added. (DOCX) [file pone.0236195.s004.docx]

| **Gene Name** | **Base Mean*** | **p. adjusted Value** | **Log2 Fold Change** | **Fold Change** |
| --- | --- | --- | --- | --- |
| Ifnb1 | 699 | 2.04E-44 | 10.84 | 1842 |
| Ccl5 | 3355 | 1.20E-75 | 10.27 | 1237 |
| Heatr9 | 171 | 3.37E-26 | 8.91 | 481 |
| Isg15 | 9917 | 5.47E-166 | 8.75 | 433 |
| Ifnl3 | 107 | 6.02E-23 | 8.49 | 360 |
| Ifi27l2a | 4508 | 1.47E-90 | 8.07 | 270 |
| Apol9a | 1676 | 8.23E-139 | 7.94 | 245 |
| Apol9b | 1386 | 1.02E-132 | 7.74 | 214 |
| Sectm1a | 1065 | 2.83E-57 | 7.47 | 177 |
| Ifit1bl1 | 1302 | 1.68E-98 | 7.39 | 168 |

**Supplementary Table 2A**

| **Gene Name** | **Base Mean*** | **p. adjusted Value** | **Log2 Fold Change** | **Fold Change** |
| --- | --- | --- | --- | --- |
| Ifnb1 | 699 | 4.83E-34 | 9.11 | 551 |
| Ccl5 | 3355 | 2.03E-61 | 8.36 | 329 |
| Ifi27l2a | 4508 | 5.81E-105 | 7.70 | 208 |
| Zbp1 | 2313 | 5.09E-156 | 7.68 | 205 |
| Heatr9 | 171 | 5.33E-21 | 7.67 | 203 |
| Lypd8 | 139 | 1.95E-27 | 7.60 | 193 |
| Isg15 | 9917 | 1.16E-157 | 7.53 | 185 |
| Oas3 | 705 | 3.53E-82 | 7.42 | 171 |
| Sectm1a | 1065 | 1.10E-68 | 7.38 | 167 |
| Apol9b | 1386 | 8.19E-139 | 7.27 | 154 |

**Supplementary Table 2B**
