# Supplementary material for: Heatr9 is an infection responsive gene that affects cytokine production in alveolar epithelial cells
Source: PLoS One. 2020 Jul 17;15(7):e0236195. doi: 10.1371/journal.pone.0236195 (PMC7367486; doi:10.1371/journal.pone.0236195)
Supplement: S2 Table — A. List of Top 10 most upregulated genes in GFP+ AEC vs. uninfected AEC comparison. Heatr9 is the third most upregulated gene in the comparison of GFP+ AEC isolated from day 3 GFP-expressing- A/Puerto Rico/8/1934 infected mice versus uninfected AEC isolated from naïve mice. Asterisk (*) to demarcate the base mean as a normalized measure of averaged reads from all samples. B. List of Top 10 most upregulated genes in GFP- AEC vs. uninfected AEC comparison. Heatr9 is the fifth most upregulated gene in the comparison of GFP-AEC isolated from day 3 GFP-expressing- A/Puerto Rico/8/1934 infected mice versus uninfected AEC isolated from naïve mice. Asterisk (*) to demarcate the base mean as a normalized measure of averaged reads from all samples. (DOCX) [file pone.0236195.s005.docx]

| **Target** | **Species** | **Assay Identifier** |
| --- | --- | --- |
| *Gapdh* | *Homo sapien* | Hs02758991_g1 |
| *Gapdh* | *Mus Musculus* | Mm99999915_g1 |
| *Ccl5* | *Homo sapien* | Hs00982282_m1 |
| *Ccl4* | *Homo sapien* | Hs99999148_m1 |
| *Mmp28* | *Homo sapien* | Hs00425232_g1 |
| *Heatr9 (C17orf66)* | *Homo sapien* | Hs00330469_m1 |
| *Heatr9* | *Mus Musculus* | Mm03015844_m1 |
| *Ifnl1* | *Homo sapien* | Hs00601677_g1 |
| *Ifna2* | *Homo sapien* | Hs00265051_s1 |
| *Ifnb1* | *Homo sapien* | Hs01077958_s1 |
| *Ifnl2* | *Homo sapien* | Hs00820125_g1 |
| *Isg15* | *Homo sapien* | Hs01921425_s1 |
| *Oas1* | *Homo sapien* | Hs00973637_m1 |

**Supplementary Table 1.**
