# Supplementary material for: Heatr9 is an infection responsive gene that affects cytokine production in alveolar epithelial cells
Source: PLoS One. 2020 Jul 17;15(7):e0236195. doi: 10.1371/journal.pone.0236195 (PMC7367486; doi:10.1371/journal.pone.0236195)
Supplement: S3 Table — In order to accurately assess gene expression in mouse and human samples, validated Taqman Gene Expression Assays were utilized from Applied Biosystems. The assay Identifier indicates which pair of primer probes were used and specifications of the specific assays. (DOCX) [file pone.0236195.s006.docx]

| **Organ** | ***Gapdh* (Ct Value)** | ***B2m* (Ct Value)** | ***Heatr9* (Ct Value)** |
| --- | --- | --- | --- |
| Heart | 18.050 | 21.105 | 33.012 |
| Kidney | 18.010 | 21.186 | 33.868 |
| Liver | 18.969 | 18.975 | 34.026 |
| Lung | 20.682 | 20.182 | 31.960 |
| Spleen | 22.435 | 21.243 | 30.531 |
| NTC | Undetermined | Undetermined | Undetermined |

**Supplementary Table 3.**
