# Supplementary figures and images for: Heatr9 is an infection responsive gene that affects cytokine production in alveolar epithelial cells
Source: PLoS One. 2020 Jul 17;15(7):e0236195. doi: 10.1371/journal.pone.0236195 (PMC7367486; doi:10.1371/journal.pone.0236195)

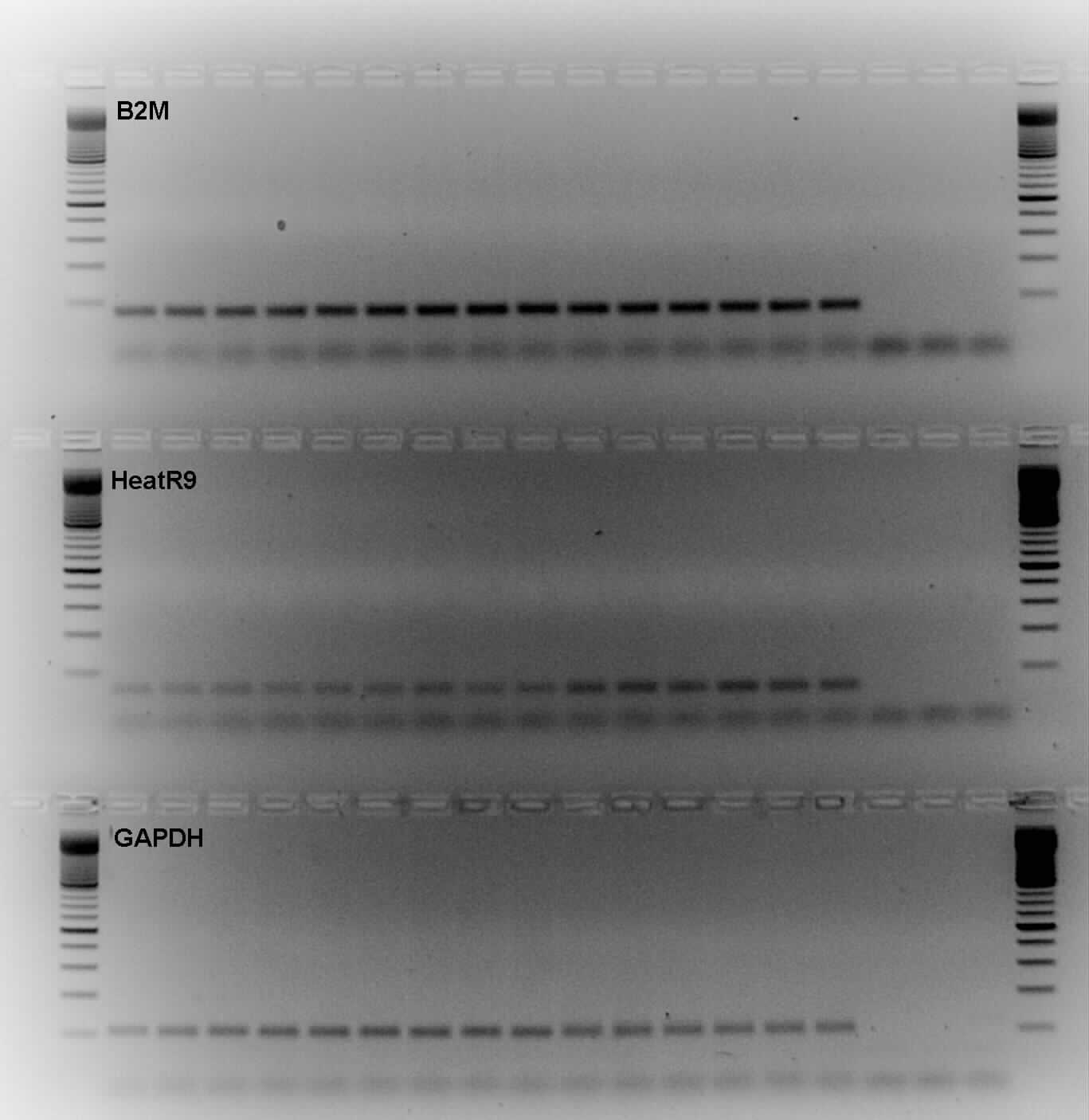


Ladder

No Template

Spleen

Lung

LIver

Kidney

Heart

Supplement: S1 Raw Data — (DOCX) [file pone.0236195.s007.docx]
